# Editorial: Advances in wild type and mutant p53 research in cancer

**DOI:** 10.3389/fmolb.2022.1064280

**Published:** 2022-11-10

**Authors:** Olga N. Hernández-de la Cruz, Guadalupe Domínguez-Gómez, Moonmoon Deb, José Díaz-Chávez

**Affiliations:** ^1^ Posgrado en Ciencias Genómicas, Universidad Autónoma de la Ciudad de México, México City, Mexico; ^2^ Subdirección de Investigación Clínica, Instituto Nacional de Cancerología, México City, Mexico; ^3^ Wellcome Trust Centre for Cell Biology, Edinburgh, United Kingdom; ^4^ Unidad de Investigación Biomédica en Cáncer, Instituto de Investigaciones Biomédicas, UNAM/Instituto Nacional de Cancerología (INCan), México City, Mexico

**Keywords:** p53, gain of function (GOF), cancer, ferroptosis, zinc ionophores, p53 reactivation therapy, *Drimia calcarata*

Four decades ago, the p53 protein was discovered (Chang et al., 1979; Lane and Crawford, 1979; Linzer and Levine, 1979), and due to its multiple functions, it has become one of the most studied transcriptional factors in humans. However, we still have an incomplete view of the molecular mechanisms regulated by p53 in normal and tumor cells. Because it is a crucial component in maintaining genomic integrity, was called “the guardian of the genome” (Lane, 1992). Even more, p53 transactivates multiple genes and joins different cellular partners, to coordinate vital cellular processes, such as cell cycle, metabolism, proliferation, cell death, and aging, among others.

The gene of p53 (TP53) is susceptible to multiple mutations, losing its suppressor tumor function but acquiring oncogenic activities called “gain of function” (GOF). Other mechanisms contributing to the structural and functional diversity of p53 include the use of alternative promoters, alternative splicing (Chen et al., 2021), and multiple post-translational modifications (PTMs) (Bode and Dong, 2004). The expression of different p53 variants in cells has significant biological and clinical implications in cancer (Rivlin et al., 2011; Alvarado-Ortiz et al., 2021).

This Research Topic on *Advances in wild type and mutant p53 research in cancer* is a Research Topic of seven articles, including two original research and five review articles, written by 39 researchers from around the world interested in unraveling the relationship between p53 and its variants in the development of cancer.

For example, under normal and stress conditions, p53 needs a precise combination of multiple PTMs to coordinate their biological functions (Liu et al., 2019; Chen et al., 2020; Zhang et al., 2022). However, the specific role of these modifications and the signaling pathways they trigger are not fully understood (Meek and Anderson, 2009). In this Research Topic, Marques et al. describe the importance of PMTs in determining the three-dimensional structure, stability, activity, and function of p53, as well as, its impact on tumor progression. In addition, PMTs can stimulate the multimerization of p53 and its mutants, forming structures known as “higher-order structures” (HOS), some of which are linked to cancer development. However, many open questions remain about their organization and function. In this context, the authors describe structural analysis methods that could help us understand the high-resolution supramolecular organization of p53 and unravel the dynamic behavior between p53 and cellular partners.

Another exciting focus is described by Ha et al. These authors detail how the structural stability of p53 strongly depends on the availability of zinc. At low zinc concentrations, p53 shows greater structural instability and lower affinity for its target promoters, thus reducing its transactivating capacity. However, some p53 mutants have a lower affinity for zinc and decreased recognition of their target sequences. Hence, a higher concentration of this metal in the medium is necessary to maintain its functional three-dimensional structure. From this perspective, the correct folding of p53 mutants in tumors could be favored by the use of metallochaperones (zinc ionophores), which could increase the permeability of tumor cells by zinc and maintain cellular homeostasis.

It has also been described that p53 regulates the expression of genes involved in the epithelial-mesenchymal transition (EMT) (Parfenyev et al., 2021; Semenov et al.). EMT is a process that stimulates phenotypic plasticity and tumor development. During EMT, cells acquire a greater capacity for invasion, extravasation, and migration, thus favoring metastasis. In this way, Semenov et al. describe the cell signaling pathways that trigger EMT, as well as, the coordinated participation of p53 with other cellular partners and miRNAs in the regulation of this process, and the impact of p53 mutants of the GOF class in the deregulation of this mechanism.

In addition, p53 participates in another poorly studied mechanism of tumor suppression: ferroptosis. Ferroptosis is a type of non-apoptotic cell death discovered in 2012; it depends on high concentrations of iron in the intracellular environment and is triggered after lipid peroxidation and an increase in the concentrations of reactive oxygen species (Kang et al., 2019; Mou et al., 2019; Liu et al., 2020; Wang et al., 2020). Babamohamadi et al. carried out an updated review on the role of p53 in ferroptosis regulation and highlighted the importance of this knowledge in the development of anticancer therapies. Further, the authors describe some p53 mutants with conformational alterations and a tendency to form intracellular aggregates, which could be related to cancer development. Moreover, they describe different types of previously reported molecules (peptides, some arginine analogs, and acetylcholine chloride) that could serve to inhibit the accumulation of these p53 mutants in tumors.

Another aspect of p53 regulation is its proteasomal degradation; in normal cells, p53 expression is negatively regulated by the E3 ubiquitin ligase MDM2 protein and its homolog MDM4 (Klein et al., 2021; Nagpal and Yuan, 2021; Pan and Blattner, 2021). The interaction of MDM2 and MDM4 with p53 masks the N-terminal region of p53, thus blocking its ability to interact with chromatin. Furthermore, MDM2, but not MDM4, promotes p53 degradation *via* the proteasome; however, in response to cellular stress, MDM2 is inactivated, decreasing its physical interaction with p53. From this perspective, some compounds blocking the interaction between p53/MDM2 and p53/MDMX complexes (such as RITA and PpIX) have been developed to restore the tumor suppressor activity of p53 and induce regression of highly malignant lesions (Jiang et al., 2019; de Bakker et al., 2022). Grinkevich et al. describe for the first time the molecular mechanism of p53 reactivation mediated by RITA and PpIX. These authors find that RITA and PpIX bind to the highly unstructured N-terminal region of p53 and induce allosteric changes that disrupt p53/MDM2 and p53/MDM4 complexes, thereby increasing the stability and function of p53. On the other hand, during Human Papilloma Virus (HPV) infections, the viral oncoprotein E6 can interact with p53 and stimulate its degradation *via* the proteasome. In this way, the virus blocks cell death and promotes its replication, eventually triggering the malignant transformation of cells (Idres et al., 2022). Interestingly, it has been reported that Human Immunodeficiency Virus Protease Inhibitors (HIV-PIs), could potentially interfere with E6-mediated proteasomal degradation of p53, thus stimulating the cell cycle arrest and apoptosis of tumor cells. In this sense, Makgoo et al. described some mechanisms of p53 restoration in HPV-positive cervical cancer. For example, using compounds that reduce E6 levels or disturb p53-E6 interactions.

Currently, there is an increase in morbidity and mortality due to different types of cancer, so the search for new therapeutic alternatives has been a primary task in recent decades. In this sense, medicinal plants constitute a biological source of hundreds of molecules with therapeutic capacities for cancer treatment (Carlos-Reyes et al., 2019; Shoaib et al., 2022). For example, Laka and Mbita reported the antitumor effects of extracts from *Drimia calcarata*, a plant widely distributed in Africa, in different human lung cancer cell lines with different Tp53 gene mutation statuses. Likewise, they analyzed the expression of genes involved in the cell cycle and apoptosis during treatment with the extracts to unravel the molecular mechanisms induced by the extracts. These studies make it possible to provide therapeutic alternatives for people who do not have access to anticancer drugs or who traditionally use natural products derived from plants to treat their diseases.

After more than 40 years of intense research, p53 continues to surprise us. In the years to come, we hope that p53 will continue to be one of our allies in the battle against cancer. This Research Topic allowed us to compile the most outstanding scientific advances in the study of the structure and function of p53 and its mutants in recent years and to describe new roles of p53 mutants in tumor initiation and progression. Likewise, we broadened the biological knowledge of p53 and its mutants, which will allow us to diversify our therapeutic strategies against cancer in the future.
